# Supplementary material for: A chromosome‐scale assembly of the model desiccation tolerant grass Oropetium thomaeum
Source: Plant Direct. 2018 Nov 15;2(11):e00096. doi: 10.1002/pld3.96 (PMC6508818; doi:10.1002/pld3.96)
Supplement: Supplementary file 1 [file PLD3-2-e00096-s001.pdf]

Reviewer #1:

This manuscript describes an improved assembly and gene model annotation of Oropetium thomaeum. The improved assembly was generated through a combination of new data types (Hi-C and RNA-seq) and new and improved assemblers. This manuscript is overall clearly written, the case for the improvement of this assembly relative to the previous assembly is strong, and the value of a chromosome level assembly for Oropetium as both a resurrection species and a Chloridoideae grass is clear.

I have one structural concern, one moderate concern, and a number of extremely minor points.

Structural:

The discussion section reads like a simple rephrasing of the abstract of the paper. In a data resource paper like this, it makes sense that there isn't much to discuss, but perhaps the editorial staff would consider allowing the omission of a discussion section or a merged discussion and results section in a data release paper like this one?

We agree with the reviewer. Given the nature of this paper, there is little to discuss without being redundant. We have restructured the paper to combine the results and discussion into one section. We feel this improves the flow of the paper and removes the previous redundancy. We have also rewritten a concluding paragraph to highlight the significance of this work and the need for more chromosome scale assemblies.

Moderate:

page 6: neither the genotype/accession ID of the oropetium plants grown for HiC, nor their relationship to the oropetium plants used for the original PacBio sequencing and/or the plants used for the RNA-seq study employed to help annotate genes is provided It would be good to include both pieces of information.

For RNAseq and Hi-C we used seeds from the Oropetium plants originally used for PacBio sequencing. Oropetium is mostly selfing with very low heterozyogisty, so we expect very few differences to be introduced between versions. We have clarified this in the methods.

It's also not clear to me if I can order oropetium seeds or cuttings -- regardless of genotype -- which would seem to be an important and necessary step in becoming a model species for the study of desiccation tolerance. <-- this last point is certainly not something I expect the authors to address in revisions to the manuscript!

Oropetium seeds are available upon request. We are happy to distribute this plant to the grass and resurrection plant communities to help establish it as a model. Oropetium is prolific and each plant can produce > 1,000 seeds. We have had issues with germination in the past, which

has prevented us from depositing the germplasm in a seedbank. We have added a sentence indicating that Oropetium seeds are available upon request.

Minor:

Page 1, line 18: I would propose striking "high quality" as it doesn't provide a lot of information content in this context. The previous assembly was of high quality relative to Illumina assemblies, of average quality relative to early Sanger assemblies, and of lower quality relative to the current v2 assembly.

We agree with the reviewer and have removed ‘high quality’. This designation is sort of arbitrary, constantly changing, and non-descriptive.

Page 2, line 43: "are polyploid" not "are polyploidy"

Corrected

Page 4, lines 120-125: the language here is a little rough. Perhaps a little more discussion/introduction of the structure of the RNA-seq dataset being employed would help. Also shouldn't it be "The expression patterns of newly annotated genes were surveyed"?

We have substantially revised this paragraph for clarity.

Page 4, line 145: why is rice in lower case but Maize capitalized?
Corrected

Page 4 lines 149-151: This also suggests that LTR retrotransposons have remained active in the lineage leading to oropetium, ruling out one potential explanation for the extremely small genomes observed in this part of the grass family tree.

We agree with the reviewer that LTRs are highly active in Oropetium and silencing is probably not contributing significantly to maintaining the small genome size. We have included one sentence in the results discussing this. However, we are hesitant to discuss this at length without more detailed analyses.

Page 5 line 180: The specific comparison of the previous assembly to sanger-based assemblies is repeated here and in the introduction. I'd suggest making it in only one of those two places. Or omitting this whole paragraph, see discussion above.

See comment above. We have removed this paragraph and replaced it with a concluding paragraph to highlight the significance of this work and the need for more chromosome scale assemblies.
